# Presentation

**Published:** 1864-12

**Authors:** 


Presentation.—Professor Charles A. Lee, of the Medical Department of the Buffalo University, was yesterday, on the occasion of the delivery of his valedictory address to the class previous to his departure for New Orleans, whither he goes for the benefit of his health, made the recipient of a cane as a token of esteem and affection on the part of his pupils. The presentation address was made by E. H. Thurston.

